# Environmental variability and modularity of bacterial metabolic networks

**DOI:** 10.1186/1471-2148-7-169

**Published:** 2007-09-23

**Authors:** Merav Parter, Nadav Kashtan, Uri Alon

**Affiliations:** 1 Molecular Cell Biology Department, Weizmann Institute of Science, Rehovot 76100, Israel

## Abstract

**Background:**

Biological systems are often modular: they can be decomposed into nearly-independent structural units that perform specific functions. The evolutionary origin of modularity is a subject of much current interest. Recent theory suggests that modularity can be enhanced when the environment changes over time. However, this theory has not yet been tested using biological data.

**Results:**

To address this, we studied the relation between environmental variability and modularity in a natural and well-studied system, the metabolic networks of bacteria. We classified 117 bacterial species according to the degree of variability in their natural habitat. We find that metabolic networks of organisms in variable environments are significantly more modular than networks of organisms that evolved under more constant conditions.

**Conclusion:**

This study supports the view that variability in the natural habitat of an organism promotes modularity in its metabolic network and perhaps in other biological systems.

## Background

Biological systems often show modularity, in the sense that they can be separated into nearly-independent structural subsystems, each of which performs a specific function [1-12]. The origin and preservation of modularity in biology is a subject of current interest [13-18].

One approach to study the origin of modularity employs computer simulations of biological evolution [19]. Such simulations make random changes to a set of networks, and select those that best satisfy a given goal. Simulations towards a constant goal usually lead to non-modular networks. Even an initially modular network rapidly evolves connections that reduce modularity but increase fitness [18]. This raises the question of which evolutionary forces can generate and maintain modular structure.

One possible force that can lead to modularity was suggested by evolutionary simulations where the goal changes over time [16,18]. Modular networks spontaneously evolved when the goal changed with time in a way that preserves the same sub-goals but in different combinations [18]. Under such 'modularly varying goals', the networks evolved and maintained modular structure, with a module for each sub-goal. When the goal changed, the connections between these modules were rapidly rewired to adapt to the new goal.

Here, we attempt to test these findings in a natural biological system, by asking whether there is a correlation between metabolic network's modularity and the variability of the environment in which it evolved. We concentrated on the metabolic networks of bacteria due to the availability and quality of the data. These networks can be systematically compared between species that live in environments that differ in their degree of variability.

Metabolic networks represent the set of biochemical metabolic reactions within a living cell [20]. Metabolic networks of diverse bacterial species have been reconstructed based on their genomic sequence and additional biochemical data [21-23]. Previous structural comparisons of metabolic networks focused on differences between kingdoms or phyla (for example, between archea and bacteria, prokaryotes and eukaryotes) [23-28]. Here, we ask whether there exists a correlation between the degree of modularity of the metabolic network of an organism and the variability in its environment.

We analyzed the metabolic networks of 117 bacteria species living in broad range of habitats including oceans, salt lakes, thermal vents, soil and within hosts. The species were classified according to the degree of variability in their environment. We measured the modularity levels and additional related structural parameters of bacteria metabolic networks, finding that the level of variability in organism's environment correlates with the modularity of the networks: The more variable the environment, the more modular the metabolic network. Our study therefore supports the view that environmental variability promotes modularity in biological networks.

## Results

### Classification of variability of bacterial environments

The natural environment of 117 bacterial species was classified based on the NCBI classification for bacterial lifestyle [29] (see methods). The classification includes six classes: ***Obligate bacteria ***[30,31] that are obligately associated with a host, either intracellulary or extracellulary. An example is *Buchnera *that lives in symbiosis inside aphids and has little contact with the outside world. ***Specialized bacteria ***that live in specialized environments such as marine thermal vents. ***Aquatic bacteria ***[32], that live in fresh or seawater environment, and are not associated with hosts. ***Facultative bacteria***, free living bacteria such as *E. coli *that often associate with a host. ***Multiple bacteria***, that live in multiple different kinds of environments such as bacteria with a wide host range, and ***Terrestrial Bacteria***, that live in the soil.

We ranked the variability of the different environments considering physical conditions (such as temperature, osmolarity, acidity, oxygen availability, etc) and heterogeneity of species [33]. *Obligate *bacteria are thought to have the most constant environment as these bacteria live within a biochemically controlled and isolated environment [34], usually with only few other species or even no other species. *Specialized *and *aquatic *bacteria are adapted to a strict realm of ecological conditions, yet their habitat is less protected and exhibits higher species heterogeneity than the *obligate *class. *Facultative *bacteria that live both in hosts and in the outside world are exposed to a more variable environment than the former classes, but less variable than the *multiple *class that spans widely different habitats. Finally, the *terrestrial *class is often considered the most variable class [35], since the soil is highly heterogeneous and has a diverse ecology.

In addition to this qualitative ordering, we sought a quantitative measure that may reflect environmental variability. One such measure is the fraction of transcription factors out of the total number of genes in the organism. The reason for using this measure is that theoretical analysis based on cost-benefit analysis suggests that transcription factors are more strongly selected in variable environments than in constant ones (chapter 10 of Ref [3]). Hence, more regulators per gene are expected the more variable the environment. It was shown [3] that the benefit of a transcription factor is highest when it can appropriately regulate gene expression in response to environmental changes, offsetting the cost of production and maintenance of the transcription factor protein and associated sensory systems [3,36]. Studies by Moran and colleagues indicate that under constant conditions, genes for transcription factors tend to be lost from the genome [37,38]. Genes of organisms in nearly constant conditions are deleted, or are constitutively expressed and do not require transcription control, as was experimentally demonstrated for *Buchnera *[39] and *Rickettsia *[40].

**Figure 1:**
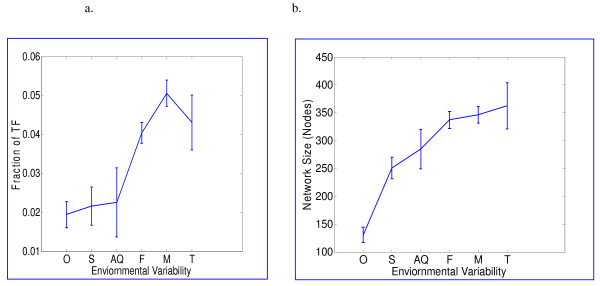
Relation between environmental variability and **a. **Mean fractional number of transcription factors out of the total number of genes in the genome. **b. **Mean metabolic network size (giant component). Error bars represent standard errors. **Abbreviation: **O-Obligate, S-Specialized, AQ-Aquatic, F-Facultative, M-Multiple, T-Terrestrial. Groups ordered along x-axis according to their predicted level of variability.

In agreement with the above, we find that the fraction of transcription factors, as well as their total number, increases with the expected variability of the bacterial lifestyle classification (Fig 1a). Note also that the size of the metabolic networks (=number of metabolites) tends to increase with the expected variability of the environment (Fig 1b).

### Modularity of metabolic networks correlates with variability in the environment

The metabolic networks of the different species were obtained from KEGG database. In the networks, each metabolite is a node and edges represent metabolic reactions (see Methods).

**Figure 2:**
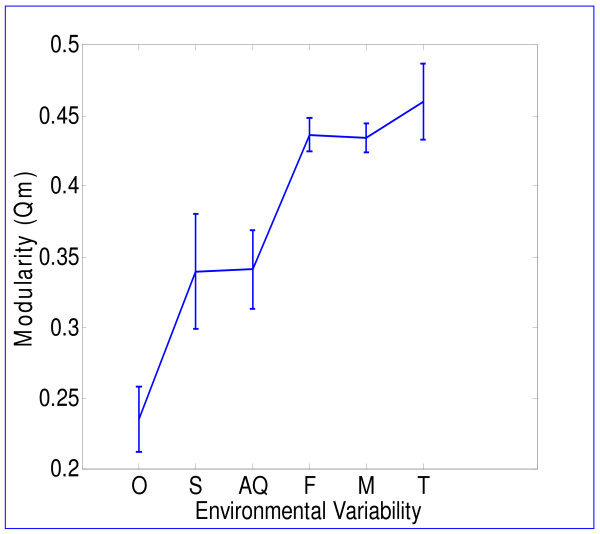
**Relation between environmental variability and modularity**. Normalized modularity measure (*Q*_*m*_) of bacterial metabolic networks versus the environmental class of the organism. Environments are ordered according to their variability ranging from O (obligate), the least variable to T (terrestrial), the most variable. Mean and standard error of *Q*_*m *_are presented for each environmental class.

We used a standard measure of modularity [18,41], to evaluate the modularity of each metabolic network. We find that modularity increases with the variability in the environment (Fig. 2). The lowest modularity is found for the *obligate *class, higher modularity is found for the *specialized *and *aquatic *classes, and the highest modularity is found for *facultative*, *multiple *and *terrestrial *classes (Correlation coefficient c = 0.63, p-value p < 10^-4^).

We further considered a control for the effects of network size on modularity, by comparing metabolic networks of the same size. This was achieved by constructing subnetworks of each network containing n = 60 nodes (metabolites), comparable to the smallest networks in the dataset. These sub-networks were constructed by contracting linear pathways and cycles, and removing dangling nodes, until a network of the required size was obtained. We find that the modularity of these equal-sized metabolic networks also significantly increased with environmental variability (c = 0.59, p < 10^-4^, additional file 2, section 1.3).

As a final control for the effect of network size, we computed the Pearson partial correlation [42] between modularity, environmental variability, and network size. We find that the correlation between modularity and variability is significant also when removing the effect of network size (c = 0.24, p = 0.02, see additional file 2 – section 1.4).

Each lifestyle class includes bacteria from different branches of the phylogenetic tree. We find low correlation between the similarity in the modularity of pairs of species and their distance on the phylogenetic tree (c = 0.1, p < 10^-4^, see additional file 2, section 3). Thus, modularity seems to be more correlated with environment than with evolutionary relatedness.

**Figure 3:**
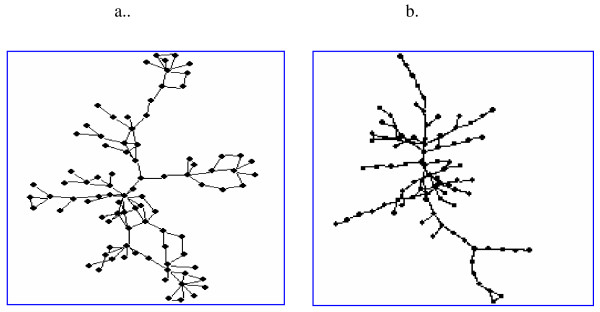
**Visualization of metabolic networks for a) *E. coli *and b) *Buchnera aphidicola***. The two networks consist of the same number of nodes (n = 89 metabolites), achieved by reducing *E. coli *network (see additional file 2, section 5).

We find that networks from species in constant environments tend to be more tree-like than those in variable environments (Fig 3). This can be quantitatively seen by considering two topological measures, betweenness-centrality [24] and cyclic coefficients [43], both properly normalized to account for different network sizes. We find that the former increases and the latter decreases the more constant the environment, suggesting tree like structures (see additional file 2, sections 2.2–2.3). The tree-like structure seems to result from a lack of alternate metabolic paths in networks from constant environments.

### Modules in organisms from variable environments are more functionally pure

We used the Newman-Girvan algorithm [41] to define structural modules in each network, and tested whether the identified modules correspond to well-defined metabolic functions [44]. A structural module in a network was considered to be also a functional module if it was enriched for substrates that had a shared metabolic function such as: central metabolism, biosynthesis of amino acids, carbohydrates, lipids etc. (according to the KEGG pathway classification [44,45]). For each network we evaluated two measures: *functionality*, defined as the fraction of structural modules that were significantly enriched for a metabolic function, and *coverage *– the fraction of biological functions that could be mapped to structural modules.

**Figure 4:**
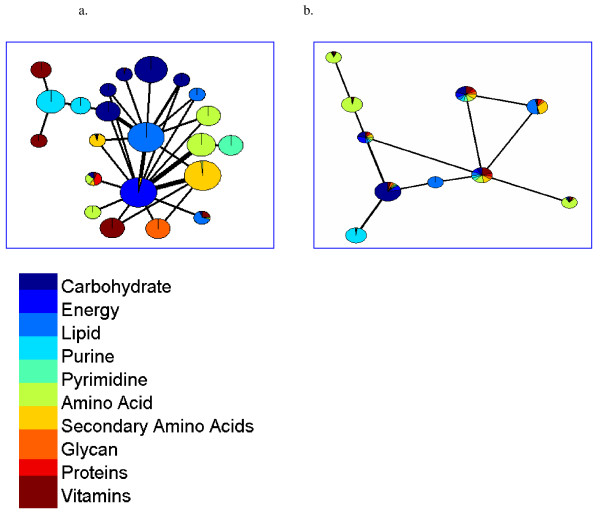
**Cartographic representation for the metabolic networks of a. *E. coli *and b. *Buchnera aphidicola***. Each circle corresponds to a structural module. Colors represent KEGG pathway classification, where the fraction of each class is proportional to the significance level of that category in the module nodes obtained from a hypergeometric test. Circle size is proportional to module's size and the thickness of edges proportional to the number of interactions between two modules. In *E. coli *most of the modules are functionally pure, and each metabolic class can be assigned to a specific structural module. In *Buchnera *network, modules are less pure and show more mixture of different functions. In three models, however, pureness of amino acid metabolism can be detected (e.g. the basis for the symbiosis with aphids)

We find that the modules in networks of organisms from constant environments usually do not correspond to a defined metabolic function, but rather to mixtures of several biological categories. In contrast, the modules of networks from variable environment classes usually corresponded to a unique function and the majority of the metabolic functions could be assigned to at least one structural module (Fig. 4 and section 4 in additional file 2).

These results further indicate that the present approach for identifying modules yields modules that have biologically significant function [44].

## Discussion

This study indicates that variability in the environment correlates with enhanced modular organization of metabolic networks, while constant environment correlate with a less modular structure.

One interpretation of these findings can be made in the context of previous simulation studies of evolution in modularly varying environments [18]. The metabolic goal that a bacterium faces can be considered as a combination of sub-goals. An example of a sub-goal is the biosynthesis of an amino acid such as histidine. If histidine is missing in the environment, the bacterium must synthesize it. If histidine is present, the bacterium can down-regulate the biosynthesis pathway and instead import this metabolite. When the environment changes over time, it introduces a different combination of such metabolic sub-goals.

Simulations suggested that varying the sub-goals leads to the evolution of networks with a modular structure, where each module corresponds to one of the sub-goals [18]. Modular structure evolves despite the fact that it is less optimal than non-modular solutions [46]. In contrast, evolution under a goal that is constant over time leads to non-modular networks, in which many nodes participate in several functions [18]. The present findings may be interpreted within this context: Bacteria that live under varying environments typically evolve a functional module for each of the varying sub-goals. Bacteria under constant conditions tend to evolve towards a less modular design.

It is interesting to note that some metabolic goals are held relatively constant even when the environment changes. An example is energy metabolism, which is needed for growth by all of the bacterial species studied, in all environments. Analysis of the metabolic networks shows that the part of the network responsible for energy metabolism (central metabolism) is less modular than other parts of the network (such as biosynthesis of amino acids, nucleotides, vitamins etc) [44,47-49]. More generally, the fraction of the metabolic network devoted to constant goals (such as central metabolism) seems to increases as the environment becomes more constant (Fig S8c in additional file 2).

**Figure 5:**
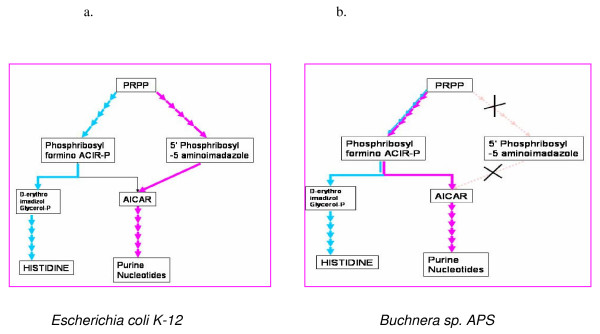
**Illustration of a mechanism that reduces modularity**. The connection between purine and histidine pathways is presented for **a**. *E. coli *and **b**. *Buchnera sp. APS*. Whereas in *E. coli *the pathways are separated, in *Buchnera *the pathways are partially combined [51].

An additional observation in the computer simulations [18] is that initially modular networks rapidly degrade into non-modular but more optimal structures when the goal becomes constant over time. Examples of such a degeneration of modularity can be seen by comparing the closely related species *E. coli *and *Buchnera*. *E. coli *lives in a variable environment, moving between its mammalian host and the external world. *Buchnera *lives in a more constant environment, as an endosymbiont of aphids. *Buchnera *is found to fuse together pathways that are separate in *E. coli*, and thus to achieve its metabolic goals with a smaller set of enzymes [50]. One example occurs in the histidine and purine modules (Fig 5). Both modules convert the metabolic substrates PRPP to AICAR in two distinct parhways. *E. coli *seems to maintain these alternative pathways because under different environments (histidine/purine rich environments) only one of the pathways is utilized. For *Buchnera*, on the other hand, as an endosymbiont that supplies amino-acids to its host, histidine biosynthesis is a fixed goal and under no regulation. Here, the purine module can count on the histidine module for AICAR production. The two pathways were thus combined into a single module, in which many of the genes are used for both functions [38,51]. It would be interesting to uncover other mechanisms that degenerate or enhance modularity by comparing networks of closely related species with different environments.

One limitation of the present study is the limited knowledge of metabolic networks for diverse species. The reconstructed networks, based on genomic data, were used to generate information about putative non-directed metabolic interactions. The present network representation ignore: i) directionality of reactions ii) reaction stochiometry iii) that only a fraction of the reactions are active under given environmental conditions (hence at best it offers only a static view on modularity). The above mentioned problems can be handled by more sophisticated network analyses [52]. Such studies employ correlated reaction sets as mathematically defined modules in biochemical reaction networks. They constitute groups of reactions in a network that always appear together in functional states of that network and therefore represent a functional module of the reaction network. Previous work has shown that these sets can include non-obvious groups of reactions and differ from groupings of reactions based on structural analyses of network topology [53,54]. One drawback of these latter approaches is that they require carefully annotated, genome-scale metabolic network, of which is only available for a handful number of species.

## Conclusion

This study indicates that the modularity of metabolic networks correlates with the variability of the environment. Such a correlation supports the view that variability in the natural habitat promotes modularity. It would be important to test this more fully as data on metabolic and regulatory networks of diverse species becomes more complete.

We currently know more about the structure of metabolic networks than about the ecology of the organisms. It is a challenge to see how far one can go in what might be termed 'reverse ecology' [36]: inferring from the structure of biological system information about the environment in which it evolved.

## Methods

### Lifestyles of bacterial species

The present classification of species can be found at additional file 1 and also at the corresponding author website. The bacterial environmental data is based on [29]. The original classification was adjusted in the present study, to better reflect environmental variability. The main adjustment was the split of the "Host Associated" class into *obligate *bacteria and *facultative *bacteria. Further corrections of specific bacteria species were performed based on literature search. For example, *vibrio *species were changed from *aquatic *to *facultative *because they are pathogens of fish. The numbers of species in the classification were as following: 35 *obligate *bacteria, 5 *specialized *bacteria, 4 *aquatic *bacteria, 42 *facultative *bacteria, 28 *multiple *bacteria and 3 *terrestrial *bacteria.

### Metabolic networks construction

Reconstructed metabolic networks of 117 bacteria were taken from the KEGG Database [45]. Each network was represented as a substrate graph, where each node corresponds to a metabolite and an edge corresponds to a reaction. Highly connected metabolites (such as ATP, NADH, H_2_0 etc) were removed – a crucial step for topological analysis [23]. Similar results were found also for a bipartite graph representation including both reactions and metabolites as nodes (additional file 2, section 1.2). Analysis was preformed on the giant connected component of the networks, to avoid bias from small isolated components. Similar results were found also when the entire network was analyzed (additional file 2, section 1.1).

### Quantitative measure of network modularity

To quantify the modularity of a network we used the normalized *Q*_*m *_measure of Kashtan et al. [18]. This measure is based on the commonly used Newmann and Girvan modularity measure *Q *[55], defined as the fraction of edges that lie within modules rather than between modules relative to that expected by chance.

A module, therefore, is as a group of nodes with many interactions between them, and few interactions to the other nodes. The algorithm attempts to find the division of nodes into modules that maximizes Q. This measure sums over all modules in the network and hence scales with network size. To allow comparison of the modularity of networks with different size and connectivity, this parameter needs to be further normalized [18]:

*Q*_*m *_= (*Q*_*real *_- *Q*_*rand*_)/(*Q*_*max *_- *Q*_*rand*_)

Where *Q*_*real *_is the Q value of the network, *Q*_*rand *_is the mean Q value of randomized networks, and *Q*_*max *_is the upper bound of *Q *for a given network ensemble. We obtained *Q*_*rand *_by averaging *Q *over hundred random networks that preserve the degree distribution of the real network [56]. *Q*_*max *_was estimated as 1 - 1/M [57] where M is the number of modules in the real network (usually the lower bound for the number of modules in the random network). For the present substrate networks, M ranged from 4 (for small networks ~100 nodes) to 22 (for large networks ~400 nodes).

## Competing interests

The author(s) declares that there are no competing interests.

## Authors' contributions

All authors designed the research and analyzed the results. MP performed the computational assays. All authors contributed to writing the manuscript.

## Supplementary Material

Additional file 2 Supplementary online material (SOM). The SOM supplies additional networks analysis, control for network size effect and descriptions for reduced networks' construction. Click here for file

Additional file 1 Microbial Environment database. Refined classification of 117 bacteria into 6 environmental classes. Click here for file
