# Supplementary material for: Environmental variability and modularity of bacterial metabolic networks
Source: BMC Evol Biol. 2007 Sep 23;7:169. doi: 10.1186/1471-2148-7-169 (PMC2151768; doi:10.1186/1471-2148-7-169)
Supplement: Additional file 2 — Supplementary online material (SOM). The SOM supplies additional networks analysis, control for network size effect and descriptions for reduced networks' construction. [file 1471-2148-7-169-S2.pdf]

Supplementary Information for:
Environmental variability and modularity
of bacterial metabolic networks

Content:

1. **Relation between modularity and variability in the environment :**

   1.1 Entire metabolic network analysis
   1.2 Reactions-substrates bipartite networks analysis
   1.3 Equal size network analysis
      1.3.1 Construction of equally-sized networks
      1.3.2 Analysis' Results
   1.4 Partial Pearson correlation between modularity and variability
   1.5 Networks Statistics

2. **Relation between variability in the environment and additional structural properties of the metabolic networks:**

   2.1 Clustering-coefficient
   2.2 Betweenness-centrality
   2.3 Cyclic-coefficient

3. **Correlations between modularity and organisms attributes**

   3.1 Single attributes analysis
   3.2 Multi way analysis of variance (n-way ANOVA)

4. Quantifying structure-function association in metabolic networks

4.1 Functionality

4.2 Coverage

5. Networks visualization for *E.coli* and *Buchnera*

5.1 Network reduction algorithm

5.2 Results

1. Relation between modularity and variability in the environment

1.1 Entire networks analysis

Figure S1

a. b.

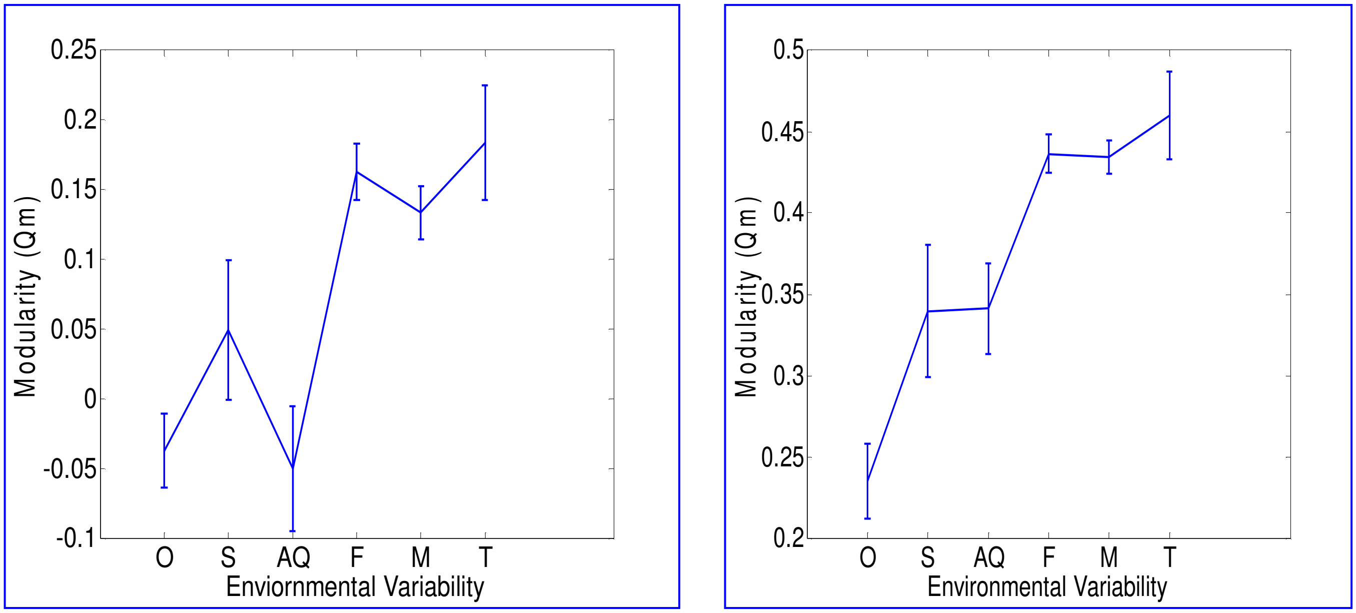

<u>Fig S1</u>: Relation between modularity and variability for **a**. Entire network c=0.55, $p<10^{-4}$.
b. The giant component of the metabolic network.

1.2 Reactions-substrates bipartite networks analysis

Reaction-substrate bipartite graph is a description of the metabolic reactions, where each metabolite node is connected to the reactions nodes that consume/produce it. Q_{rand} was computed by averaging over bipartite random networks that preserve the metabolites' as well as the reactions' degree distribution.

Figure S2

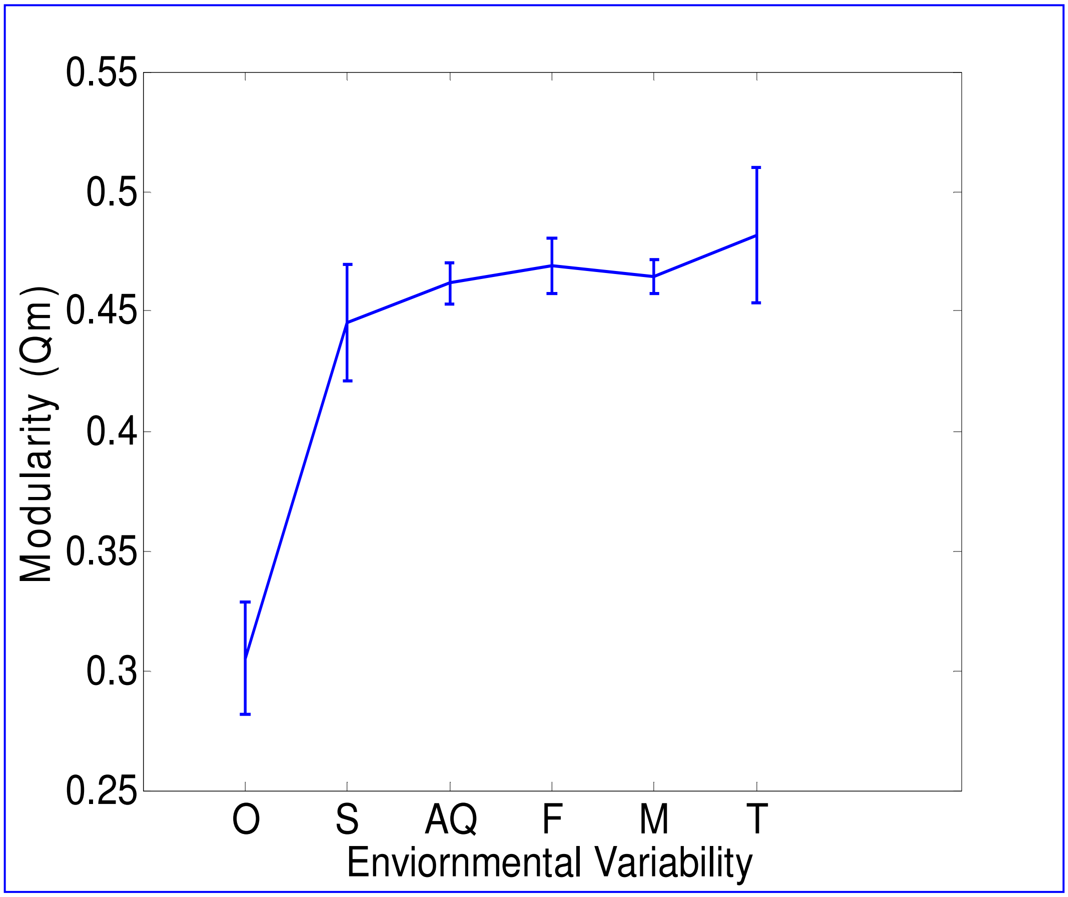

Fig S2: Relation between modularity and variability in bipartite metabolic networks, c=0.59, $p<10^{-4}$.

1.3 Equal size network analysis

1.3.1 Construction of equally-sized networks

Metabolic networks of bacteria with different lifestyles are of different sizes. To control for the effect of the network's size, we repeated the analysis on reduced networks with the same number of nodes. We constructed a set of equal size networks (60 nodes) by a serial removal of nodes with degree <=2 (i.e. we remove cycles and shorten linear pathways).

1.3.2 Analysis Results

Figure S3

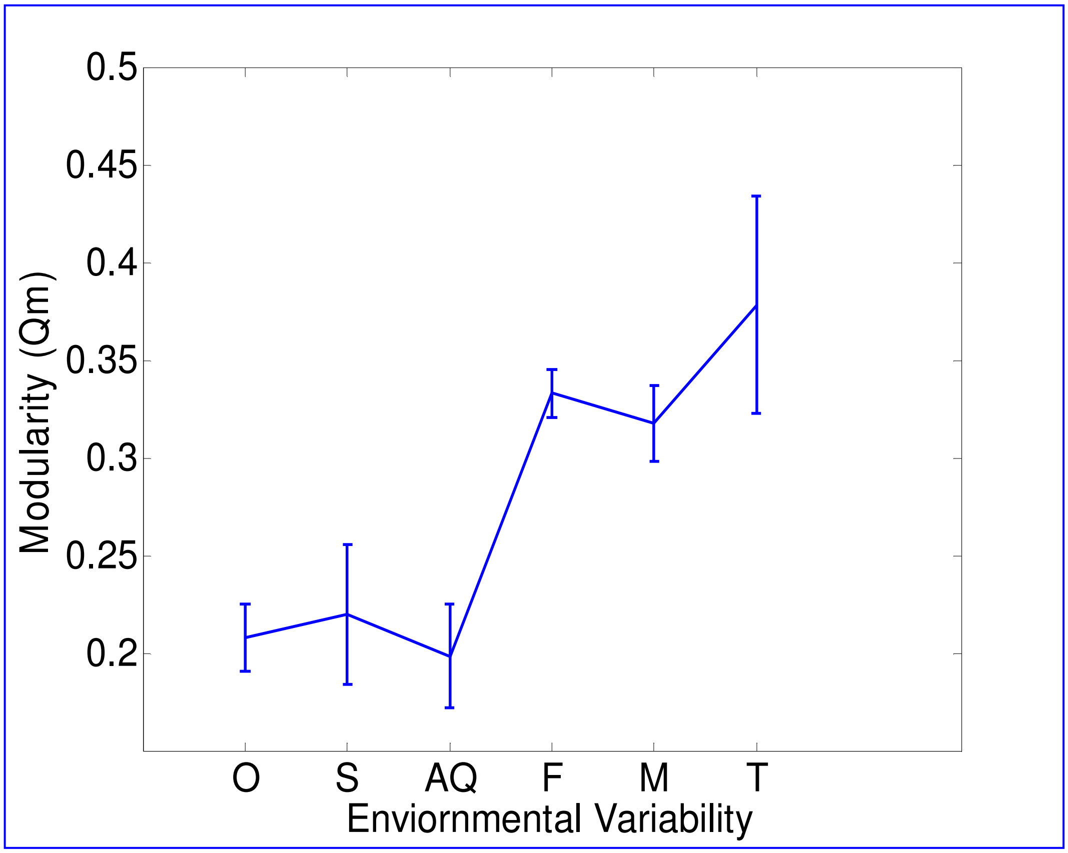

Fig S3: Modularity-variability relation in equal sized networks with 60 nodes (metabolites), c=0.5, $p<10^{-4}$.

1.4 Partial Pearson correlation between modularity and variability

Perason's partial correlation between X and Y conditioned on Z allows one to compute the correlation between X and Y, discounting the correlations between X and Z and between Y and Z [1]. We computed the correlation between modularity and variability conditioned on the size of the networks (=number of metabolites). For this purpose, we grouped the species into two classes corresponding to bacteria with low-variability lifestyle (*Obligate*, *Specialized* and *Aquatic*) and bacteria with high-variability lifestyle (*Facultative, Multiple, Terrestrial*). The partial Pearson correlation between modularity and variability conditioned on the size of the network is c=0.24 with p-value= 0.02. This implies that the correlation is still significant when conditioned on network size. It is not as high as the full correlation coefficient because variability itself seems to correlate with network size (Fig 1b, main text).

1.5 Networks Statistics

**Network Statistic**	**#Nodes (Full Net)**			**#Edges (Full Net)**			**#Nodes (Giant Component)**			**#Edges (Giant Component)**			**Qm (on the giant component)**		
	**min**	**median**	**max**	**min**	**median**	**max**	**min**	**median**	**max**	**min**	**median**	**max**	**min**	**median**	**max**
**Obligate**	**136**	**330**	**712**	**99**	**281**	**596**	**21**	**89**	**280**	**23**	**102**	**322**	**-0.2**	**0.26**	**0.5**
**Specialized**	**481**	**496**	**521**	**428**	**451**	**460**	**206**	**235**	**299**	**222**	**258**	**336**	**0.22**	**0.36**	**0.43**
**Aquatic**	**510**	**577**	**714**	**450**	**500**	**655**	**224**	**264**	**386**	**251**	**290**	**440**	**0.28**	**0.3365**	**0.41**
**Facultative**	**230**	**694**	**856**	**171**	**630**	**811**	**40**	**333**	**473**	**42**	**385**	**574**	**0.11**	**0.4529**	**0.54**
**Multiple**	**448**	**690**	**878**	**398**	**660**	**835**	**193**	**454**	**360**	**214**	**431**	**553**	**0.26**	**0.45**	**0.5**
**Terrestrial**	**618**	**710**	**743**	**559**	**677**	**706**	**282**	**385**	**421**	**329**	**463**	**498**	**0.41**	**0.47**	**0.5**
**Total**	**136**	**562**	**878**	**99**	**515**	**835**	**21**	**282**	**473**	**23**	**322**	**574**	**-0.2**	**0.4141**	**0.54**

2. Relation between variability in the environment and other structural indices of the metabolic networks

2.1 Clustering-coefficient

Clustering coefficient reflects the local community structure [2]. The clustering-coefficient of a node is defined as the number of edges between its neighbors divided by the total number of possible such edges (considering the network as undirected). The clustering coefficient of the network is the mean over all nodes with degree >1. We computed the clustering coefficient for each natural network and its corresponding set of random networks. The normalized measure was obtained by dividing the measure of the real network by the mean of the random networks. That normalized clustering coefficient significantly increases with the variability of the environment (Fig. S4a).

2.2 Betweenness-centrality

Centrality of node X is defined as the number of shortest paths between pairs of nodes in the network that go through X. Betweenness centrality measures the average centrality of all nodes in the network. We scaled this parameters by dividing it by the maximal value that could be obtained for a network of the same size [3]. Analytic analysis of this measure reveals that networks with a tree like topology have higher betweenness than networks with cycles. The intuition behind this is that for acyclic graph, each non leaf node must be visited when traveling from one side of the tree to the other side. When adding shortcuts to the tree, one creates alternative pathways and the centrality of the node decreases. We find that the normalized betweenness-centrality is anti correlated with variability (Fig. S4b).

2.3 Cyclic-coefficient

The cyclic coefficient of a node is defined as the inverse of the mean of the shortest loop length connecting it with each pair of its neighbors [4]. Cyclic coefficient of a network is the mean over all nodes with degree>1. Networks without cycles, such as perfect trees, have cyclic coefficient of zero. Generally, tree like networks are characterized by low cyclic coefficient. We normalized this parameter by the mean of its value for randomized networks.

We find that the cyclic coefficient correlates with the variability of the environment (Fig. S4c).

Figure S4:

a.

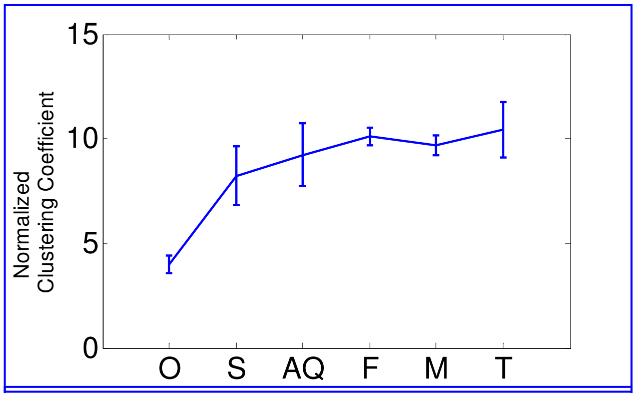

b.

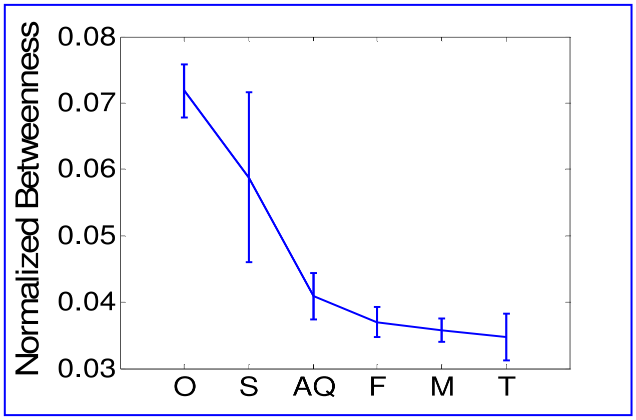

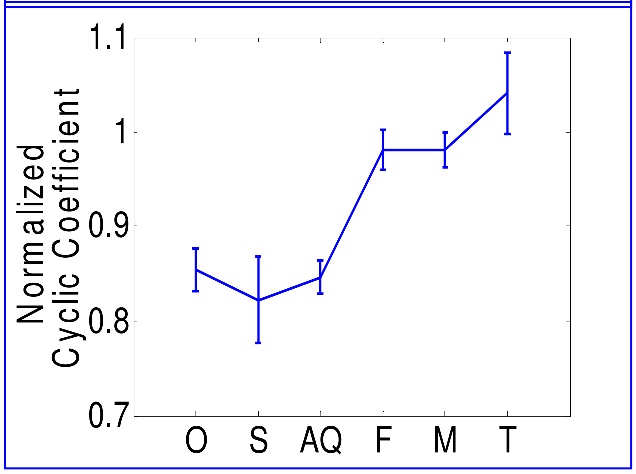

Fig S4: Topological indices as function of variability in the environment:

- **a.** Normalized clustering-coefficient CC(Real)/CC(Rand) . c=0.62, p< 10^{-4}
- **b.** Normalized betweenness-centrality c= -0.66 , p< 10^{-4}
- **c.**. Normalized cyclic-coefficient. c=0.41, p< 10^{-4}

3. Correlations between modularity and organisms' attributes

3.1 Single attributes analysis

We considered a certain set of possible factors that can explain the modularity level of organism's metabolic network:

1. Total gene number
2. Number of transcription factors
3. Fraction of transcription factors out of all genes
4. Phylogenetic relations

   The phylogenetic distance between two organisms was defined as the length of the path between them on the phylogenetic tree. The latter was constructed base on the KEGG DB: http://www.genome.jp/kegg/catalog/org_list.html

Figure S5:

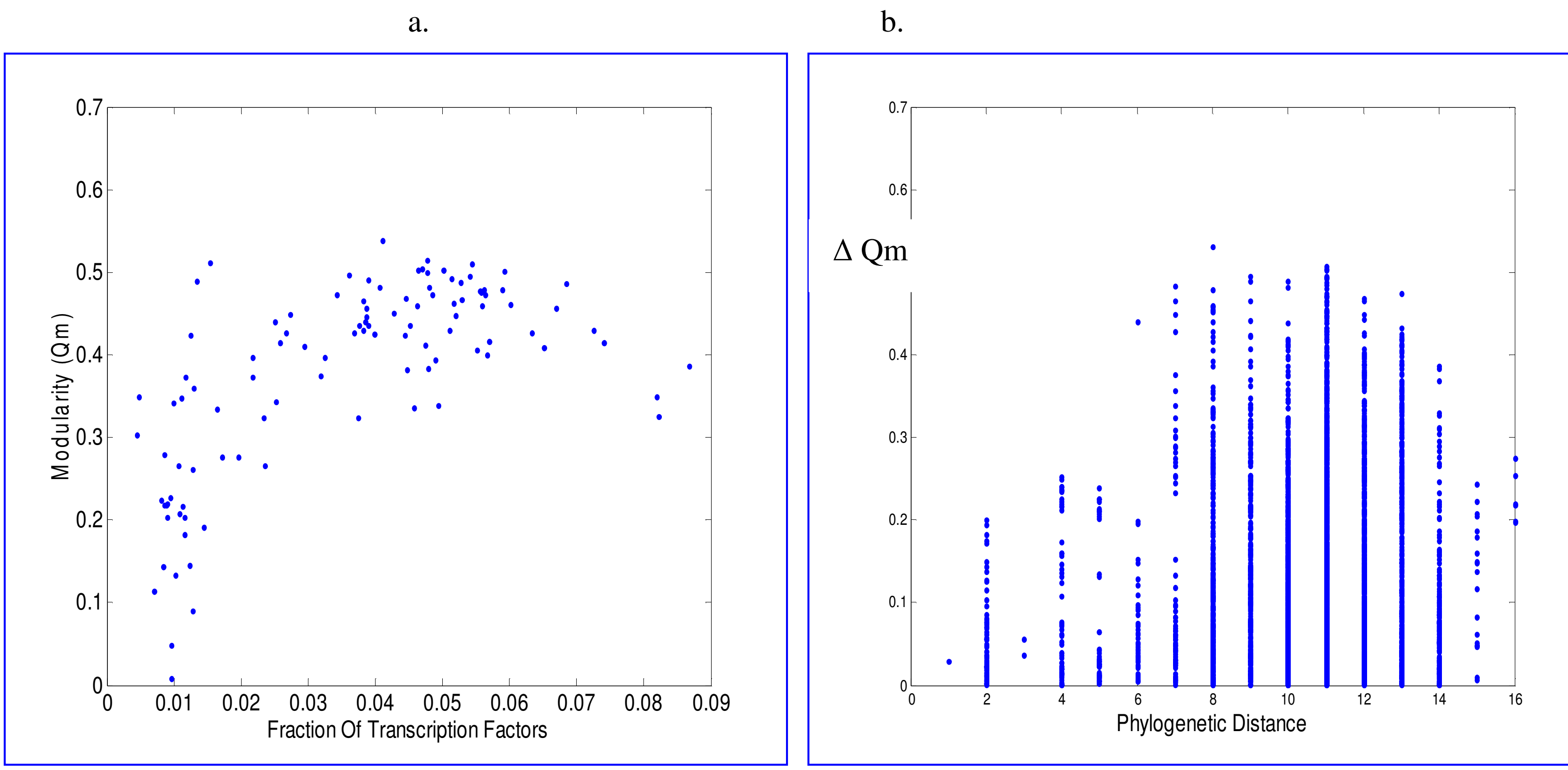

Fig S5: Relation between modularity (Qm) and **a**. fraction of transcription factors, **b**. phylogenetic relatedness.
b. Each dot corresponds to a pair of bacteria. Its Y axis corresponds to the difference in Qm and its X axis corresponds to the evolutionary distance between these bacteria.

Table S1: Summary of Univariate Analysis

Correlation Coefficient (R)	Fraction of Transcription Factors	Total number of Transcription Factors	Total Number of Genes	Phylogenetic Distance
Qm	**0.65**	0.62	0.51	**0.1**

3.2 Multi way analysis of variance (n-way ANOVA)

Multi way analysis was performed in order to evaluate the quality of each presumably explanatory factor while taking into account the effects of all other variables on the response (e.g. Δ Qm). Since the phylogenetic distance is an attribute of a pair, we perform pairs analysis. That is, for each pair we consider 5 measures: Δ#(Partial)TF, Δ #Genes, Phylogenetic distance and Δ Qm. Using the Matlab function anovan, one obtains a p-value for each explanatory variable. The lower the p-value, the stronger is its association to the response variable. To obtain a distribution of p-values we performed a bootstrap procedure, where we sampled with repetitions 100 samples for 1000 iterations. We find that the partial number of transcription factors out the total number of genes is the best predictor of modularity. Genome size is a less powerful predictor, and phylogenetic distance is a weak predictor.

We also evaluated the influence of each attribute on the environmental variability (Fig S7).

Figure S6: P-values histogram for modularity prediction

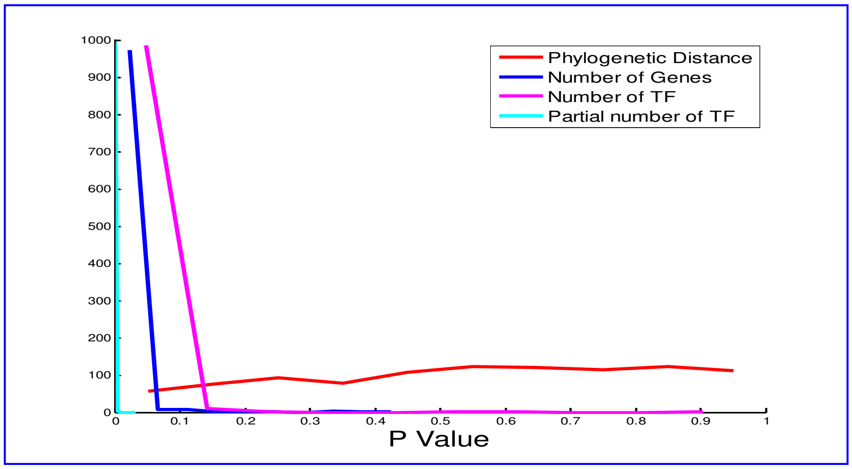

<u>Fig S6</u>: P-values histogram of ΔQm explanatory variables. Sample size equals 100; histogram was obtained from 1000 samples.

Figure S7: P -values histogram for variability prediction

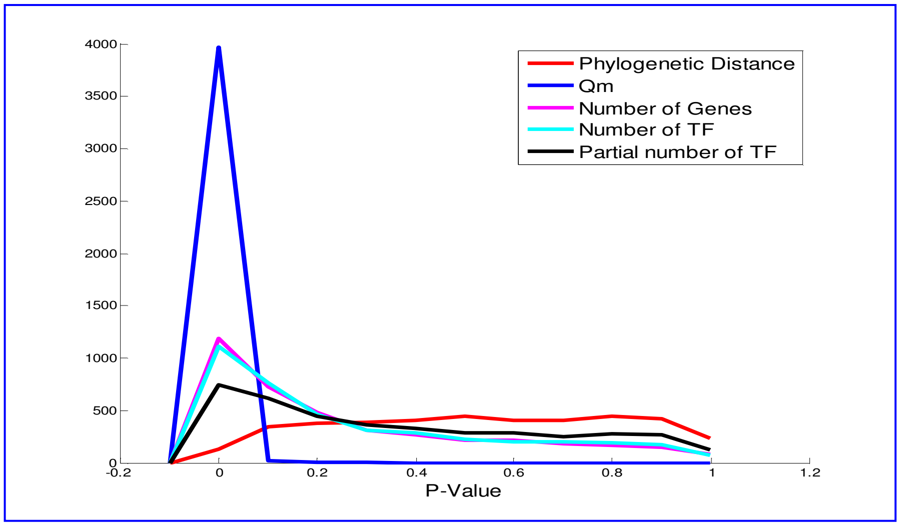

Fig S7: P-values histogram of environmental variability explanatory variables. Sample size equals 100; histogram was obtained from 1000 samples.

4. Quantifying structure-function association in the metabolic networks

We tested the structural modules, obtained from the Newmann-Girvan algorithm, for enrichment in metabolic functions. We score the strength of <Structure, Function> association by evaluating two measures:

- **Functionality** –The fraction of structural modules that are significantly enriched in at least one metabolic function.
- **Coverage** – The fraction of metabolic functions that are found to be enriched in at least one structural module in the network.

We found that these quantities correlate well with the variability in the environment.

Fig S8: Functionality and Coverage as a function of environmental variability

a.

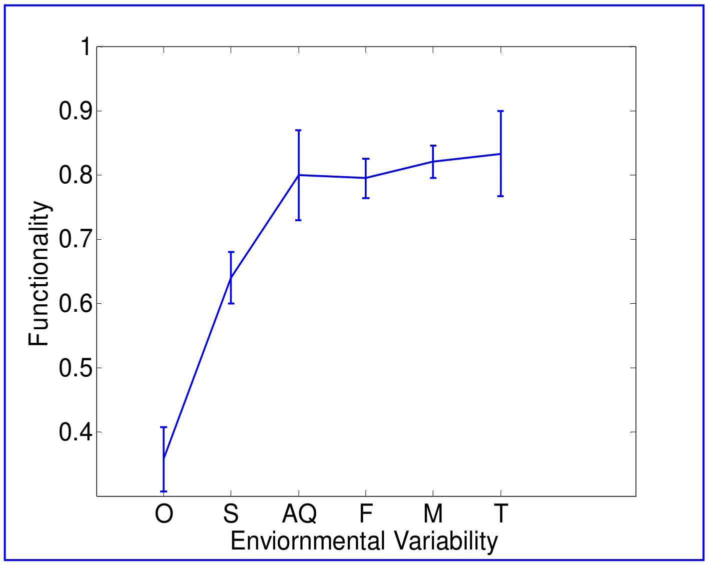

b.

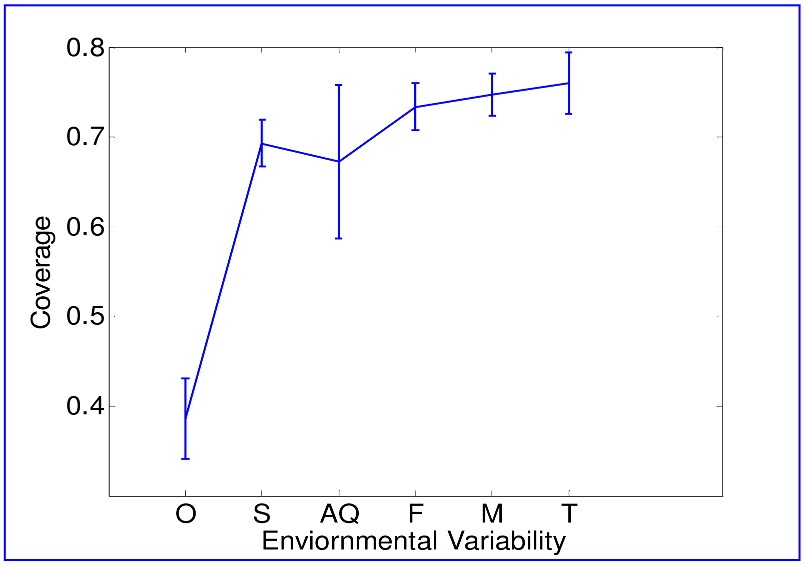

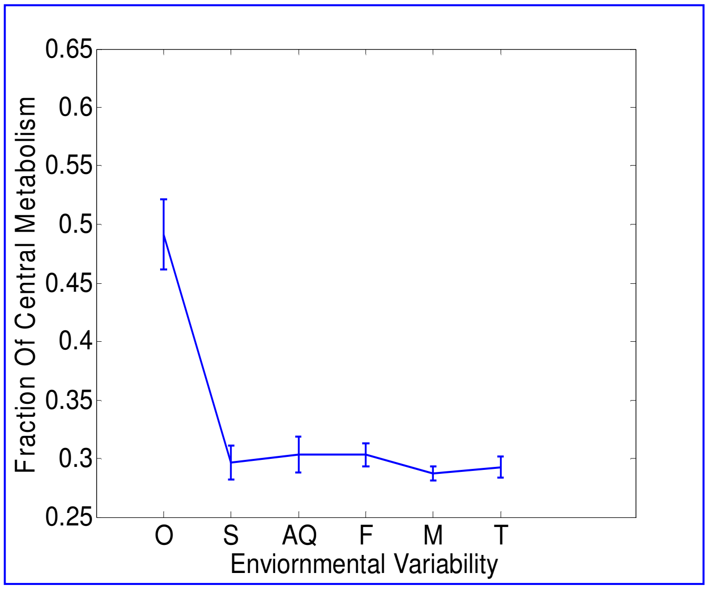

Fig S8: **a**. habitat variability (x axis) and the correspondence of structural modules to metabolic functions (y axis) c=0.66 ,$p<10^{-4}$. **b**. habitat variability (x axis) and the correspondence of metabolic functions to structural modules (y axis) c=0.6 1,$p<10^{-4}$. **c**. The fraction of the central metabolism module nodes out of the total number of nodes in the metabolic network is presented as a function of environmental variability.

5. Networks Visualization for *E.coli* and *Buchnera*

To help understand the structure of different networks, it is useful to obtain an image of the networks, in which the networks are of the same size. We therefore reduced a “varying environment” network in a manner that preserved its original topological properties. This step enables comparison of two equally sized networks from two different environmental groups (Fig S9a,b). Please note that the procedure described below is meant only for ease of visualization, and is distinct from the

reduction procedure defined in section 1.3.1 that was used for comparison of structural indices.

5.1 Description of the procedure for metabolic network reduction for visualization

Motivation:

Given two networks: *G1*: = (*V1*, *E1*), *G2*: = (*V2*, *E2*) where *V1*>*V2*, *E1*>*E2*. We wish to compare these networks by eye with respect to stractural properties.

As we saw, topological comparison between two different size networks involves normalization of the structural indices (over random network ensemble). Though this is simple computationally, capturing the difference by eye is less intuitive. As a preceding step we then wish to reduce the larger network (*V1*) by removing *V1*-*V2* nodes and yet to preserve the information embedded within the original network with respect to structural as well as functional properties. Although a common procedure for connected sub network sampling, 'breadth-first-search' (BFS) is applicable for some applications that involve analysis of local structural properties such as clustering coefficient and network motifs [5], it is not applicable when analyzing global properties (i.e. modular organization). As an example, lets consider the network of varying environment bacteria, composed of ~20 modules. We would like to remove 80% of its nodes. If we perform BFS from different starting points, the resulted network will usually correspond to 1-3 modules of the original network and thus will not reflect the metabolic capabilities of the original network as well as its pronounced modular design. In what follows we describe our method that uses a special attribute of metabolic networks: its hierarchical modular organization that reflects both the structural and the functional aspects we would like to maintain.

Reduction Procedure:

In the present context we want to compare two networks of related bacteria with different lifestyles. The varying environment network (of *E.coli*) composed of 473 nodes while constant environment network (of *Buchnera*) has only 89 nodes.

- Problem Generalization:

  We can generalize the question in the following manner:

Given a network *G*: = (*V*, *E*), *Q (G)* =*Q**

Construct a network G': = (*V'*, *E'*), *Q(G')*=*Q**

where

- $V' \subset V$
- $E' \subset E$
- $E'/V' \sim E/V$

That is, we wish to construct subnetwork that will preserve the modular organization (*Q**) of the complete network (Constrain 3 will be fullfilled by our construction).

According to the Newman and Girvan approach, the modularity score of a network (*Q*) is the summation over the strength of its modules. That implies that we can reduce our problem of network reduction to a problem of reducing a module while preserving its strength (where module strength is defined as its contribution to its Q measure). Formally, given a module Mi(Vi, Ei) with strength Qi, we need to build a module Mi'(Vi',Ei') , $Vi' \subset Vi$, $Ei' \subset Ei$ such that Qi'=Qi . This procedure ensures that Q is preserved since $Q=\sum Qi$, thus it preserves the modular organization of the complete network. A module's strength can be considered as the ratio between edges within the module to edges that connect it to other modules. This intuitive definition is the key for the present construction. It is easy to see that we can maintain (at least approximately) the ratio of number of edges inside the module to the number of edges outside the module, for each module, and by that obtain a smaller network with the same community structure.

Procedure ReducedNetSameQ(G,S)

{

```
    // G(V,E) - network to be reduced
   // S = target size, S<|V|

    G'={}  //Reduced network initialization
    S'={}  // Module size vector of the reduced network
     1.   (Q,M)=ComputeModularity(G)
                //M:  M(i , j)=1 if node j belongs to module i
               // M (i): list of nodes in module i
               // Q: array of modules strength, ∑Q(i)=Q*
    2.    for i=1,….,Nm      //Nm= number of modules
    3.        S'(i)=|M(i)| *S/|V|   // Compute the size of the reduced module
    4.        Gi=GetSubNet(M(i),G) // the induced subgraph over the module's nodes.
    5.        (Gi',Lout(i))=ReduceModuleSameQi(G,Gi,M(i),Q(i), S'(i),S(i))
    6.        G'=G' ∩ Gi';
    7.        (G'')=ConnectModules(Lout, G')
                // Connect the modules (Gi' subnetwork) allowing Lout(i) edges for
                module i ,this  needs to be relaxed when the connections are between
                modules  of different sizes. The criterion for choosing the edges is
                based on the distances between the reduced modules.
    8.        G'=G' ∩ G'';
    9.   Return G'
}
```

Procedure ReduceModuleSameQi (G, Gi, Mi, Qi, Si', Si)

{

```
        1.      Lin=|Gi| //number of links (edges) within the module
        2.      Lin'=Lin * Si'/Si;
        3.      Lout=E(Mi)-2*Lin
        4.      Lout'= Lout * Si'/Si ;
        5.      Gi'=SelectSubNet(Gi,Lin',Si')  //returns reduced module i.
                with Si' nodes and Lin' edges
```

```
6.      Return Lout(i),Gi'
        // Gi' within module edges
        //  Lout(i) number of allowed out edges for module i
}
```

Procedure SelectSubNet (Gi,Lin',Si')

```
{
1. Nodes={}
2. Nodes(1)=FindCenterNodeInModule(Gi)
3. for i=2,….Si
4.        Nodes(i)=ClosestNodeToModule //choose the closest node to
                                         the nodes already selected
5. Mi'=GetSubNet(Nodes,G) //the induced sub-graph over the selected nodes
6. Select Lin' edges from Mi'
7. If |Mi'|<Lin'
8.        add Lin'-|Mi'| edges between closest nodes in Mi'
9. Return Gi'

}
```

Practically, it may be hard to obey all the constrains imposed by the algorithm, and yet our experience suggests that even if some of the local constrains are relaxed the resulting network seems to exhibit very similar hierarchical organization as the original one. Future work may employ Monte-Carlo optimization approaches to satisfy this problem.

5.3 Results

Figure S9:

a. b. c.

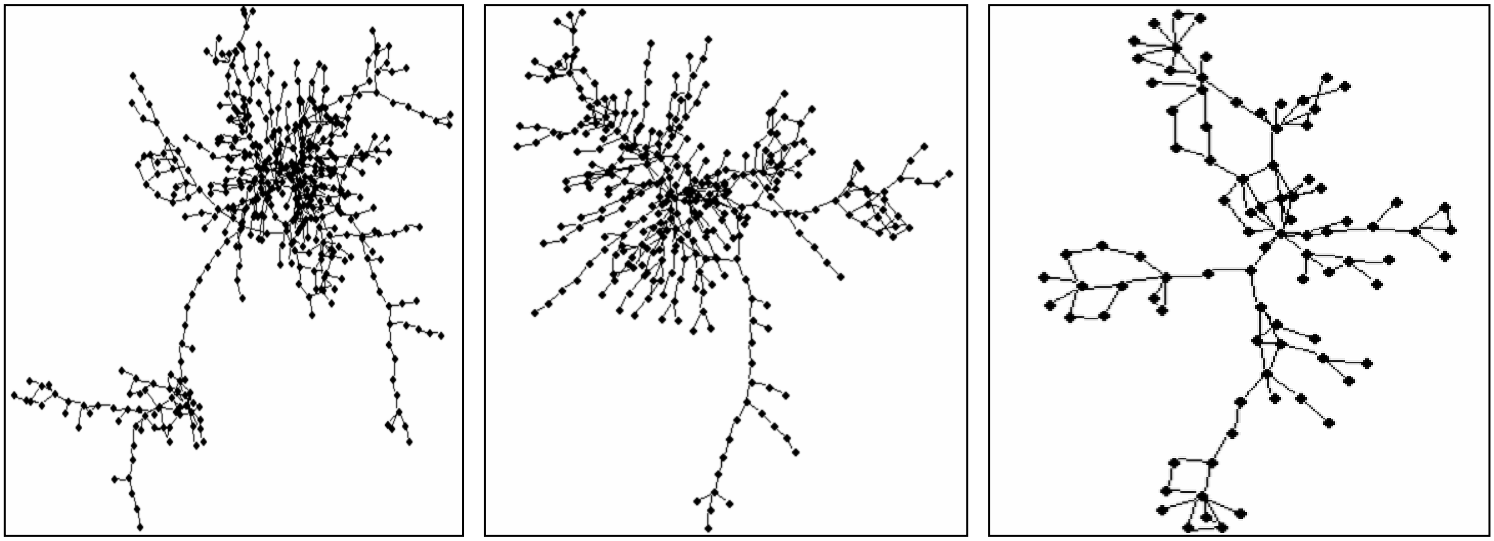

Fig S9: Reduction of a network composed of the ten largest modules in the *E.coli* network (connected subgraph, ~80% of the original network).
a. Full network (giant component), 473 nodes (Metabolites), 575 edges (Reactions).
b. Giant-component composed of only the 10 largest modules of the network, 330 Nodes, 407 edges.
c. Reduced network 89 Nodes, 114 Edges.
The reduced network (c) has a similar modularity score as the original network (Qreal~0.8).

References

1. Cramer D: **A cautionary tale of two statistics: Partial correlation and standardixed partial regression**. *Journal of Psychology* 2003, **137(5)**:507-511.
2. Watts DJ, Strogatz SH: **Collective dynamics of 'small-world' networks**. *Nature* 1998, **393**:440-442.
3. Zhu D, Qin ZS: **Structural comparison of metabolic networks in selected single cell organisms**. *BMC Bioinformatics* 2005, **6**:8.
4. Kim HJ, Kim JM: **Cyclic topology in complex networks**. *Phys Rev E Stat Nonlin Soft Matter Phys* 2005, **72**:036109.
5. Milo R, Shen-Orr S, Itzkovitz S, Kashtan N, Chklovskii D, Alon U: **Network motifs: simple building blocks of complex networks**. *Science* 2002, **298**:824-827.
